# Endothelial Aldehyde Dehydrogenase 2 as a Target to Maintain Vascular Wellness and Function in Ageing

**DOI:** 10.3390/biomedicines8010004

**Published:** 2020-01-03

**Authors:** Ginevra Nannelli, Marina Ziche, Sandra Donnini, Lucia Morbidelli

**Affiliations:** 1 Department of Life Sciences, University of Siena, 53100 Siena, Italy; gine_nannelli@hotmail.it (G.N.); sandra.donnini@unisi.it (S.D.); 2 Department of Medicine, Surgery and Neurosciences, University of Siena, 53100 Siena, Italy

**Keywords:** endothelial cells, oxidative stress, inflammageing, endothelial dysfunction, aldehyde dehydrogenase-2, cardiovascular disease, neurovascular disease

## Abstract

Endothelial cells are the main determinants of vascular function, since their dysfunction in response to a series of cardiovascular risk factors is responsible for disease progression and further consequences. Endothelial dysfunction, if not resolved, further aggravates the oxidative status and vessel wall inflammation, thus igniting a vicious cycle. We have furthermore to consider the physiological manifestation of vascular dysfunction and chronic low-grade inflammation during ageing, also known as inflammageing. Based on these considerations, knowledge of the molecular mechanism(s) responsible for endothelial loss-of-function can be pivotal to identify novel targets of intervention with the aim of maintaining endothelial wellness and vessel trophism and function. In this review we have examined the role of the detoxifying enzyme aldehyde dehydrogenase 2 (ALDH2) in the maintenance of endothelial function. Its impairment indeed is associated with oxidative stress and ageing, and in the development of atherosclerosis and neurodegenerative diseases. Strategies to improve its expression and activity may be beneficial in these largely diffused disorders.

## 1. Introduction

We searched PubMed from its inception up to December, 2019, using the terms “ALDH2, endothelial cells, endothelial dysfunction, endothelial senescence, ageing, oxidative stress, inflammageing, cardiovascular diseases, neurovascular diseases” to identify publications in English that described the mechanism of action of ALDH2 activity in vascular function, preclinical evidence of beneficial effects of ALDH2 expression/activity for endothelial function, or clinical evidence of the benefit of ALDH2 activity modulation in cardio- or neuro-vascular diseases. We mostly selected publications from the past 10 years that we judged were relevant, but we did not exclude widely referenced and highly regarded older publications.

### 1.1. Endothelial Function and Dysfunction

Vascular function such as the heartbeat is an essential system for the good functioning of our body. Under normal conditions, endothelial cells (ECs) in blood vessels, through the release of vasoactive and anti-aggregatory mediators and the functioning of antioxidant systems, regulate blood pressure, protecting from hypertension and atherosclerosis. They also constitute a blood barrier, preventing leukocyte infiltration and inflammation into the vascular wall and surrounding tissues [1,2].

Conversely, endothelial dysfunction has been identified as a hallmark of most cardiovascular diseases. Dysfunction of ECs is correlated with an imbalance in the production of key regulators of the vascular homeostasis such as nitric oxide (NO) and growth factors, and/or impaired activity (uncoupling) of endothelial NO synthase (eNOS), associated with increased reactive oxygen species (ROS) levels and vascular oxidative stress [3,4]. Inflammatory factors such as inteleukin-6 (IL-6), tumor necrosis factor-α (TNF-α), intercellular adhesion molecule 1 (ICAM-1) and loss of the antioxidant mechanism are among the most important causes of vascular dysfunction [5].

When endothelial dysfunction occurs, the ability of endothelium to perform one or all of these functions is decreased. In this condition, ECs switch to a pro-inflammatory profile, characterized by loss of barrier integrity, and reduced release of vasoactive molecules associated with increased release of pro-thrombotic mediators [2,5]. Our studies and those of others showed that endothelial dysfunction is linked to impaired endothelial cell survival and physiological angiogenic outcomes with a later rearrangement of the microcirculation that contributes to the onset of various diseases [6,7,8]. Both anti- and pro-angiogenic therapeutic strategies have been developed for treating human diseases. While angiogenesis inhibitors have been shown to have success in many diseases as cancer, few treatment protocols with the aim to stimulate angiogenesis in ischemia-associated diseases have reached the clinic. Indeed, since endothelial function has been proposed as “barometer for cardiovascular risk”, identification of the molecular determinants underlying endothelial integrity and functionality is a medical need [5,6,9,10,11]. In this scenario, risk factors for endothelial dysfunction are represented by pathological conditions as hypertension, diabetes and atherosclerosis, and lifestyles as high-fat diet, tobacco smoke, alcohol intake, and physical inactivity [12]. Since endothelium is a regulator of exchanges between the vascular wall and surrounding tissues, it is not surprising that dysfunctional ECs can lead to the impairment of other tissues [2,6,12]. Indeed, assessment of vascular function and structure, and in particular of endothelial dysfunction appears to play a crucial role in a broad array of human diseases as cardiovascular and neurodegenerative diseases, tumor growth and metastasis [2,5,8,9,10,11,12].

### 1.2. Endothelial Senescence

Cardiovascular diseases (CVDs) represent the major cause of disability and death in the elderly population of the Western World. As introduced above, a large number of risk factors plays a role in the development of CVDs, especially ageing which is associated with well-characterized phenotypes [8,13,14]. Over time, aged blood vessels become stiffer and thicker, and their ability to release vasoactive mediators, particularly NO, decreases, while vascular permeability increases, associated with the process of mild vessel inflammation, increased vessel thickness and compromised angiogenic response [14,15,16,17,18]. Of note, even if age remains the main determinant of vascular senescence, healthy vascular ageing can be achieved, and endothelial function is a key element of the heathy vasculature.

Senescent vascular endothelium and other tissues are frequently characterized by chronic mild inflammation [12,13]. In particular, the age-related low grade, chronic, and systemic inflammation is indicated by the term “inflammageing” [12,13]. The indicators of inflammageing include elevated levels of inflammatory mediators such as IL-1, IL-6 and TNF-α, which characterize many age-related pathological phenotypes. These indicators derive from an imbalance of the immune response (immunosenescence) [19].

A large population of senescent cells in organs has the potential to negatively impact organ renewal capabilities and functions, especially on ECs, accelerating the onset of several age-associated pathologies, as CVDs and cancer [15,20].

However, senescent cells may also exert various positive effects on individuals. As senescence is associated with irreversible growth arrest, it is considered to be a tumor suppressor process [21]. In particular, indirect evidence indicates that endothelial senescence plays a causal role in microvascular rarefaction and affects the ability of cells to proliferate and form capillary-like structures [20]. The timely and challenging issue for modern biomedical sciences focused on ageing is the identification of the biological targets and the pharmacological tools able to promote “healthy ageing”.

### 1.3. Aldehyde Dehydrogenase-2 (ALDH2)

The aldehyde dehydrogenase (ALDH) gene superfamily encodes enzymes that are mainly responsible for the irreversible oxidation of various aldehydes. Three major classes of mammalian ALDHs (classes 1, 2 and 3) have been identified. Classes 1 and 3 contain both constitutive and inducible cytosolic isozymes. Class 2 consists of constitutive mitochondrial isozymes [22,23]. ALDH2 is primarily involved in the detoxification of acetaldehyde, the first intermediate of ethanol metabolism, into acetate, a less active byproduct [23]. In addition to acetaldehyde, other aldehydes are metabolized by ALDH2 [23,24]. The sources of aldehydic substrates of ALDH2 can be endogenous or exogenous. Endogenous reactive aldehydes are generated as byproducts of cell metabolism and include malondialdehyde, 4-hydroxynonenal (4-HNE), DOPAL, and acrolein [23]. Exogenous aldehydes are found in industrial and environmental pollutants and may be produced during metabolism of xenobiotics. Among these, acrolein, acetaldehyde and formaldehyde are the major reactive species found in tobacco smoke and car exhaust [23,24]. Generally, aldehydes are toxic molecules that react rapidly with amino acid residues, e.g., thiols to form the genotoxic DNA- and protein-adducts in cells [23,24].

ALDH proteins are found in all cellular compartments, including cytosol, mitochondria, endoplasmic reticulum and the nucleus. Some proteins have more than one cellular location. Nevertheless, ALDHs also catalyze some reactions involved in the formation of bioactive molecules that regulate important physiological functions. This is the case of some ALDHs as ALDH1A1, 1A2 and 1A3 that convert retinal aldehyde in retinoic acid. In turn, retinoic acid acts as a ligand for retinoic X receptor (RXR) and nuclear retinoic receptor (RAR), participating in a number of growth and developmental processes [23,25,26].

ALDH2 arises as an important gatekeeper of ROS overproduction that a cell is able to tolerate. In fact, the main function of mitochondrial ALDH2 is to protect mitochondria and cells from the damaging effect of aldehydes, by oxidizing the substrates into their corresponding non-toxic carboxylic acids. Some studies distinguish between direct and indirect anti-oxidative properties of ALDH2. The direct anti-oxidative properties are assumed to depend on its potent reductase function with its highly activated sulfhydryl groups [27]. In addition to oxidative capabilities, ALDH2 possesses nitrate reductase activity responsible for the bioconversion of nitroglycerin to 1,2-glyceryl dinitrate (GDN), thus inducing NO release [28,29,30]. ALDH2 protein can be the substrate of various post-translational modifications, including oxidation, S-nitrosylation, phosphorylation, nitration, acetylation, glycosylation, and adduct formation, most of which reduce its activity [31].

In the present review, we describe the effects of oxidative stress-linked accumulation of aldehydes in the cells, focusing on ECs, and discuss the contribution of ALDH2 in several endothelium-dependent disorders, including senescence.

## 2. ALDH2 and Endothelial-Related Diseases

**Figure 1:**
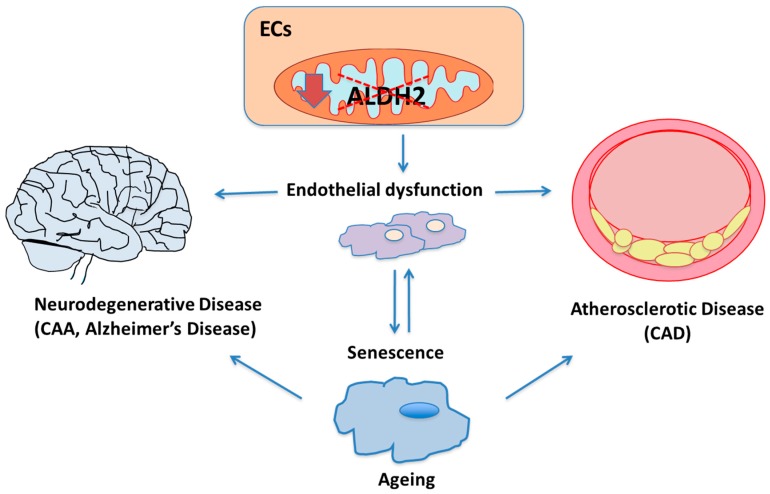
The role of aldehyde dehydrogenase-2 (ALDH2) in endothelial function and endothelial-related diseases. ALDH2 downregulation (down arrow) and/or catalytic inactivation (dashed red lines) into mitochondria in endothelial cells (ECs), leading to inefficient aldehyde metabolism, is recognized to affect endothelial functions and make senescence occur faster. Senescence itself might further aggravate endothelial dysfunction. In turn, ALDH2 is believed to be involved in the ageing process, in particular ageing related-cardiovascular diseases, such as atherosclerosis and coronary artery disease (CAD), and affect the cerebrovascular unit, contributing to the etiology of neurovascular degenerative diseases, including Cerebral Amyloid Angiopathy (CAA).

Different diseases have in common EC impairment and dysfunction and more precisely an alteration in ALDH expression and enzymatic activity, in turn responsible for changes in oxidative status and inflammation (Figure 1).

### 2.1. ALDH2, Oxidative Stress and Ageing

Advanced age is an independent risk factor for life-threatening diseases, including coronary artery disease, stroke, and neurodegenerative diseases, which are directly related to ageing-associated EC dysfunction [32]. Despite senescence being studied deeply for many centuries and many steps in our understanding of it accomplished, the process of ageing remains largely difficult to remedy and remains, unfortunately, inevitable [33]. In addition to the “inflammageing” hypothesis, many theories have been proposed to explain the process of ageing [33]. In particular, the general free radical theory of ageing indicates that ageing is caused by ROS-dependent accumulation of damage [34]. In other words, the traditional view in the field of free radical biology is that free radicals and ROS are toxic and able to directly damage a large plethora of biological targets. Furthermore, the accumulation of damage leads to the process of ageing resulting in various diseases [35]. Recent findings revealed that the accumulation of toxic aldehydes plays a key role in ischemia-reperfusion injuries and in ageing as much as ROS from which they derive [36,37,38,39].

Accordingly, some studies support the close link between ALDH2 dysfunction and ageing, especially in the heart [40,41]. In particular, ALDH2 has been reported to protect ECs against oxidative stress events due to aldehydes, and cell senescence [41,42].

One of our recent works showed that inhibition of ALDH2 activity negatively impacted EC function. We demonstrated that ALDH2 silencing or inhibition significantly affected cell proliferation, migration and altered cellular permeability, in terms of reduced VE–cadherin and ZO-1 expression at cell–cell contacts and increased cellular permeability in human umbilical vein endothelial cells (HUVECs) [42]. Although the mechanism of action has not been fully elucidated, our results indicate that ROS production and 4-hydroxy-2-nonenal (4-HNE) accumulation, a secondary end-product of lipid peroxidation, contribute to mediate endothelial dysfunction and the onset of senescence in siALDH2 and daidzin-treated cells [42]. These findings were supported by the observation that exposure to the ROS scavenger, N-acetylcysteine, affected the pattern of 4-HNE adducts, reduced ROS and recovered cell survival in siALDH2 cells [42]. The aldehyde 4-HNE is a reactive molecule which forms adducts with proteins, lipids and DNA. Excessive 4-HNE adduct formation has been reported in ischemic cardiovascular tissue isolated from rodents and humans [43,44]. From a bioenergetic standpoint, we observed that ALDH2 attenuation reduced basal and maximal respiration and abolished mitochondrial reserve capacity in ECs [42]. Intriguingly, this latter finding suggested that ALDH2 supports mitochondrial bioenergetics by increasing basal, maximal and consequently reserve capacity. Further, the changes in cell morphology, from polygonal to an enlarged and irregular shape, evoked the senescent phenotype and this led us to explore further details. The analysis of typical senescent markers, such as p21/p53 expression and SA-β-gal, further suggested that siALDH2 or daidzin-treated ECs present early signs of senescence [42]. This scenario suggests a protective role of ALDH2 on endothelium. The inhibition of ALDH2 affects endothelial functions, bioenergetics and metabolism, and makes the acquisition of a senescent phenotype faster. The acquisition of a senescent phenotype might have a defensive role, but the expansion of senescent cells might itself further aggravate the damage and accelerate ageing.

From a mechanistic point of view, the accumulation of endogenous reactive aldehydes, including 4-HNE, and inhibition of ALDH2 by these reactive species increased cell vulnerability to aldehyde-induced damage [45]. Indeed, susceptibility to lipid peroxidation byproducts of ALDH2 enzymatic activity was reported [46]. It was observed that ALDH2 is inactivated by aldehydes at low concentrations (<10 μM) and ROS. ALDH inactivation may interfere with the detoxification of lipid aldehydes, promoting their accumulation, which, in turn, is known to trigger ROS production, amplifying oxidative damage in the cell. Specifically, in ECs inactivation of ALDH2 correlates with vascular damage, vasoconstriction, and thrombosis [47].

Moreover, evidence showed the role of acetaldehyde in tissue and cell damage. In particular, a study reported that ALDH2 prevented acetaldehyde-related toxic effects by alleviating oxidative stress and apoptosis in HUVECs [47]. For instance, it has been observed that in HUVEC, ALDH2 over-expression protects cells from the toxic effects of exposure to different concentrations of acetaldehyde. As a result, the generation of ROS is decreased, as well as apoptosis and activation of stress signaling molecules, such as signal-regulated extracellular kinases (ERK1/2), and the p38 mitogenic activated MAP kinase protein [47]. Of note, the acetaldehyde-induced ROS generation, apoptosis and activation of stress molecules were prevented by the ALDH2 transgene in a manner similar to antioxidant alpha-tocopherol, indicating that facilitation of acetaldehyde detoxification by ALDH2 transgene overexpression is able to counteract acetaldehyde-induced EC injury and activation of stress signals. These data consecutively highlight the therapeutic potential of ALDH2 in the prevention of cell damage induced by ethanol consumption [47], features of which are very similar to those of accelerated ageing.

Consistently, mutations in ALDHs genes as well as their downregulation or ALDHs catalytic inactivation, leading to inefficient aldehyde metabolism, may contribute in the etiology of various diseases including cardiopathies and neurovascular degenerative diseases, including the cerebral amyloid angiopathy (CAA) and Alzheimer’s disease (AD) [24,48].

### 2.2. ALDH2 and Atherosclerosis

Heart failure is a clinical manifestation characterized by alterations in cardiac morphology and functions that lead to reduced cardiac output and/or elevated intra-cardiac pressure at rest or during stress. Coronary artery disease (CAD), diabetes mellitus and alcohol abuse are common determinants of myocardial diseases, which result in ischemic injury, metabolic disorders or toxic damage [49]. Nevertheless, advanced ageing leads to alteration in morphology and function of the heart in the absence of other accompanying cardiovascular risk factors. In particular, ALDH2 is believed to be involved in the ageing process and ageing related-cardiovascular diseases. In fact, although ALDH2 is better known for its involvement in ethanol metabolism, it is also crucial for cardioprotection through the detoxification of reactive aldehydes such as 4-HNE and the bioconversion of nitrates into NO [27,29]. ALDH2 is largely seen as a critical enzyme involved in protecting the heart from ischemic injury. Much evidence, including meta-analysis, have assessed the associations between ALDH2 rs671 (ALDH2*2) polymorphism inactivating the enzymatic activity, and CAD [50]. Additionally, results from various studies conducted on Asian patients have highlighted the strong association between myocardial infarction and ALDH2 rs671 polymorphism [51]. In this context, the majority of studies focused on myocardium [52], with limited attention on ECs or vessels. Nonetheless, ALDH2 activity is impaired by oxidized low-density lipoproteins (ox-LDLs), possibly by post-translational modifications. In particular, ox-LDLs are found to exert an inhibitory effect on ALDH2 activity by preventing mitochondrial sirtuin 3 (SIRT3) expression [31]. SIRT3 is one of the members of the sirtuin family, which have a common core domain and an important role in ageing, stress resistance and metabolic regulation. In particular, SIRT3 has received much attention for its role in mitochondrial metabolism and ageing [53].

A study corroborates the association between ALDH2 and SIRT3 [54]. This study strengthens the concept that moderate ethanol consumption is associated with a positive effect on eNOS activation that results in antiatherogenic actions. In particular, Xue and colleagues showed that in human aortic ECs (HAECs), low-dose ethanol resulted in SIRT3 inactivation, leading to rapid activation of ALDH2. ALDH2 activity mediates ethanol-induced eNOS activation and prevents ROS accumulation [54]. Thus, ALDH2 activation promoted by SIRT3 inactivation improves HAEC function, resulting in an anti-atherogenic effect. Consistent with this finding, another recent study showed that ALDH2 silencing or inhibition aggravated the atherosclerotic process. In ApoE^−/−^ mice the silencing of the ALDH2 gene was associated with a severe inflammation of the vascular wall and the formation of larger and more unstable plaques [55]. In-vitro experiments with HUVECs further illustrated that inhibition of ALDH2 activity resulted in elevated inflammatory molecules, an enhanced nuclear translocation or phosphorylation of pro-inflammatory transcription factor such as NF-κB or AP-1 involved in vessel permeability and plaque development [55]. Of note, in ECs, we and other reported that several natural agents such as polyphenols of extra virgin olive oil modulate, beyond ROS production, the expression and activity of enzymes and transcription factors involved in atherogenesis [56,57]. These data support the assumption that metabolic cell redox state has a marked impact on the cell transcriptome and proteome, and, in turn, on cell functions. In this context, it would be of interest to know the effects of nutraceuticals on ALDH2 activity in controlling vessel integrity.

Overall, these data convincingly indicate that ALDH2 silencing or inhibition exacerbates the atherosclerosis process, increasing plaque development and vulnerability with aggravated inflammation. Nevertheless, further work is needed to fully understand the clinical value of ALDH2 and its activation in the prevention or treatment of atherosclerotic diseases, such as CAD.

### 2.3. ALDH2 and Neurodegenerative Diseases

Alzheimer’s disease (AD) is classified as a progressive neurodegenerative disorder, characterized by neuropathological changes in particular brain regions and in a variety of neuro-transmitter systems. The progressive neuronal degeneration that occurs in AD generally leads to dementia, the most common consequence, affecting cognitive functions in patients such as memory, thinking and reasoning [58]. Unfortunately, AD is becoming more prevalent as human lifespan increases. AD is a multifactorial disease driven by a combination of genetic and environmental factors and can be divided in two forms: familial and sporadic cases [59]. A characteristic feature of AD is the deposit of amyloid β peptides (mainly Aβ1-40 and Aβ1-42) in the brain that form extracellular amyloid plaques. These plaques lead to neuronal dysfunction, cell death, and loss of synaptic connections, notably due to the ensuing inflammation and oxidative stress [60]. Increased oxidative stress is reported to play a critical role in the pathogenesis of AD prior to the onset of Aβ deposition and cognitive impairment. Cerebrovascular dysfunction has emerged as a critical feature of neurodegenerative diseases [61]. Cerebral amyloid angiopathy (CAA), a cerebrovascular disease that is frequently associated with AD, is characterized by the accumulation of Aβ in cerebral microvessels. In CAA, the endothelial dysfunction is thought to alter Aβ homeostasis and to promote infiltration of the brain parenchyma with circulating toxic molecules [61]. At the same time, Aβ peptides, especially the vasculotropic isoform Aβ1–40, affect brain blood vessels, altering their fundamental functions, including impairment of vasoactive tone and barrier functions, vascular remodelling as well as suppression of the intrinsic angiogenic properties of endothelium [48,62,63,64]. Although CAA remains clinically distinct from AD, their common features have the potential to link cerebrovascular and neurodegenerative pathways in the ageing brain. The product 4-HNE is formed during oxidative stress, and is able to react with many molecules such as proteins, and to accumulate in the brain. Many studies have shown that 4-HNE covalently modifies Aβ via 1,4 conjugates, altering the function of Aβ and impairing cellular features such as metabolism, cell signaling and structural integrity [65]. Since ALDH2 protects mitochondrial functions through the detoxification of 4-HNE that accumulates in this organelle, it is not surprising that recent epidemiological studies showed a correlation between ALDH2*2 loss-of-function mutations in Asian patients and a higher incidence of AD in these people [24,45].

One potential intervention for the treatment of AD can be to reduce the toxicity caused by the Aβ peptides, particularly Aβ1-40. Previous studies from our lab elucidated the role of ALDH2 in the protection of endothelium against Aβ1-40 damage. In particular, the chronic exposure of cultured EC to Aβ results in profound modifications of cell pro-angiogenic functions, shifting their phenotype toward the senescence program and reducing its pro-angiogenic capability [66]. Aβ peptides also increase intracellular 4-HNE in ECs by impairment of mitochondrial ALDH2 activity [48]. Consistently, in an ALDH2^−/−^ knockout null mouse model, mice exhibited both neuronal and vascular pathological changes associated with AD. In fact, mice exhibited progressive, age-related cognitive deficits in non-spatial and spatial working memory and other features, together with a multitude of AD-associated signs, including 4-HNE adducts as well as age-related increase in Aβ [67].

In addition to the observed AD-like changes in the brain, significant vascular alterations were found in cerebral microvessels (CMVs) of ALDH2^−/−^ mice. In comparison to the wild type, CMVs of these mice displayed marked increases in HNE adducts and age-related increases in monomeric Aβ. Moreover, ALDH2^−/−^ mice exhibited endothelial dysfunction, and increased Aβ deposits in microvessels [67].

From these studies it seems that ALDH2 activity may play a critical role for preserving endothelial function in cerebrovascular units and preventing age-related dysfunctions.

## 3. Conclusions

Global average lifespan is increasing as a result of many factors, such as lifestyle. But, as longevity continues to increase, ageing-related diseases and, consequentially, the need for new therapeutic interventions arise. Today, regenerative medicine plays a significant role relative to other therapies, including those for the treatment of CVD.

In this review we showed that ALDH2 asserts a protective role in the endothelium against different types of stressors including age-associated dysfunctions. ALDH2 has emerged in recent years as a crucial guardian against several stressor or toxic insults, supporting its potential role in many diseases, including cardiovascular and cerebrovascular diseases that are linked to ageing (Table 1).

Since senescence-related endothelial dysfunction plays a crucial role in the pathogenesis of several diseases, further studies are necessary to fully elucidate the role and protective mechanisms of ALDH2 in the endothelium.

## Figures and Tables

**Table 1:** Summary of main findings of ALDH2 and ageing, atherosclerosis or neurodegenerative diseases.

ALDH2 Status	Tissue/Cells	Molecular Mechanism	Function	Ref.
**ALDH2 and vascular ageing**				
ALDH2 activation	Heart (rat)	(−) aldehydic adducts (carbonylation)	(−) cardiac dysfunction	[28]
ALDH2 activation	Liver (rat)	(−) ROS (−) pro-inflammatory cytokines (−) 4-HNE and MDA	(−) tissue apoptosis (+) mitochondrial membrane potential	[30]
ALDH2 transgenic mice	Heart	(+) ROS (−) AMPK phosphorylation (−) SIRT1	(+) ageing-induced cardiac hypertrophy (+) apoptosis (+) mitochondrial injury	[40]
ALDH2 activation	Heart (mouse)	(−) 4-HNE-protein adducts (−) protein carbonyls (+) autophagy flux (+) SIRT1	(+) cardiac function	[41]
ALDH2 gene silencing	Endothelial cells	(+) ROS (+) 4-HNE	(−) respiration (+) senescence	[42]
ALDH2 gene transfection	Endothelial cells	(−) ROS	(−) apoptosis (−) ERK/p38-MAPK	[47]
**ALDH2 and atherosclerosis**				
ALDH2*2 loss-of-function	---	(+) ox-LDLs	(+) coronary artery disease	[50,51,52]
ALDH2 gene silencing	ApoE^−/−^ mice	(+) ROS	(+) vessel wall inflammation (+) plaque instability	[55]
ALDH2 activation	Endothelial cells	(−) ROS; (+) eNOS (−) SIRT3 activation	---	[54]
ALDH2 inhibition	Endothelial cells	(+) NF-κB (+) AP-1	(+) permeability (+) plaque formation	[55]
**ALDH2 and neurodegenerative diseases**				
ALDH2*2 loss-of-function	Brain	---	(+) incidence of Alzheimer’s disease	[45]
ALDH2^−/−^ mouse model	Brain	(+) 4-HNE adducts (+) Aβ	(+) cognitive deficits (+) endothelial dysfunction (+) Aβ in microvessels	[47]
ALDH2 inactivation	Endothelial cells	(+) 4-HNE	(−) angiogenesis (+) senescence	[48]
